# Strength training promotes attenuation of fatty liver while improving insulin resistance and inflammatory biomarkers in obese rats induced by high-fat diet

**DOI:** 10.1590/1414-431X2025e14505

**Published:** 2025-08-22

**Authors:** S. Torezani-Sales, A.P.L. Damiani, A.R. Madureira, J.P. Cordeiro, G.H. Taufner, B.V. Nogueira, M.C. Dias, M.M. Sugizaki, A.S. Leopoldo, A.P. Lima-Leopoldo

**Affiliations:** 1 Programa de Pós-Graduação em Nutrição e Saúde, Centro de Ciências da Saúde, Universidade Federal do Espírito Santo, Vitória, ES, Brasil; 2 Programa de Pós-Graduação em Ciências Fisiológicas, Centro de Ciências da Saúde, Universidade Federal do Espírito Santo, Vitória, ES, Brasil; 3 Programa de Pós-Graduação em Educação Física, Centro de Educação Física e Desportos, Universidade Federal do Espírito Santo, Vitória, ES, Brasil; 4 Departamento de Morfologia, Centro de Ciências da Saúde, Universidade Federal do Espírito Santo, Vitória, ES, Brasil; 5 Instituto de Ciências da Saúde, Universidade Federal de Mato Grosso, Sinop, MT, Brasil

**Keywords:** Metabolic dysfunction, Physical exercise, Hepatic homeostasis, Inflammatory cytokines, Visceral adiposity

## Abstract

Obesity is an independent risk factor for metabolic dysfunction-associated steatotic liver disease (MASLD). Non-pharmacological strategies, such as strength training (ST), have been investigated for their effectiveness in attenuating MASLD. This study evaluated the effects of ST on hepatic fat accumulation in an experimental model of obesity. Thirty-day-old male Wistar rats (∼150 g) were assigned to either a standard diet (SD) or a high-fat diet (HFD). The experimental protocol lasted 26 weeks and was divided into two phases: 1) obesity induction and maintenance (16 weeks) and 2) ST intervention (10 weeks). After the 16th week, HFD-fed rats were further divided into sedentary obese (Ob) and obese trained (ObST) groups. The ST protocol consisted of 4-5 vertical ladder climbs with 60-s intervals, three times per week, using 50-100% of maximal load. Body weight (BW), fat pads, total body fat (BF), adiposity index (AI), and muscle strength were assessed, as were glycemic, lipid, inflammatory, and histological parameters. ST reduced BW, epididymal and visceral fat depots, triglycerides, total cholesterol, glucose, leptin, and tumor necrosis factor (TNF)-α levels while improving insulin resistance. In conclusion, ST significantly attenuated hepatic steatosis in obesity, promoting metabolic and anti-inflammatory benefits. These findings suggest that ST may be an effective therapeutic strategy for MASLD, and further studies are needed to elucidate its molecular mechanisms and clinical applications.

## Introduction

Obesity is a multifactorial chronic disease characterized by excess adiposity (1). According to the World Health Organization, the prevalence of obesity has almost tripled since 1975, reaching 16% of the adult population over 18 years of age in 2022. The growing prevalence of obesity may reflect changes in dietary and behavioral patterns, marked by a strong increase in the intake of hypercaloric diets and physical inactivity (1). Obesity can be associated with several pathological disorders, such as metabolic dysfunction-associated steatotic liver disease (MASLD), also known as non-alcoholic fatty liver disease (NAFLD) (2).

NAFLD is characterized by fat accumulation (or hepatic steatosis) in more than 5% of hepatocytes. Presently, new criteria for the diagnosis of MASLD include evidence of fatty liver assessed by biopsy, imaging, or blood biomarkers, in addition to necessarily being overweight/obese, presence of type 2 diabetes mellitus (T2DM), or evidence of metabolic dysregulation (3). Excess fat accumulation in the liver occurs mainly due to an increase in adipose tissue lipolysis, an elevation in hepatic *de novo* lipogenesis, an increase in intake of high-fat diets (HFD), as well as a reduction in lipid β-oxidation (4).

AT hypertrophy promotes the activation of macrophages in obesity that act in the production and secretion of cytokines (5). In this context, chronic inflammation promotes increased signaling and release of pro-inflammatory cytokines, such as tumor necrosis factor-alpha (TNF-α), interleukin (IL)-1β, IL-6, and IL-8 (6), which are associated with increased insulin resistance (5,6). The state of insulin resistance associated with obesity may contribute to increased hepatic lipogenesis and lipotoxicity, leading to accumulation of lipids in hepatocytes (7).

Non-pharmacological approaches such as physical exercise have been extensively studied for their role in preventing and treating ectopic fat deposition in the liver (8,9). Baek et al. (8), using hypercaloric diet-fed animals subjected to aerobic and strength training (ST) for 8 weeks, demonstrated that both exercise protocols effectively reduced intrahepatic triglyceride levels, even in the absence of significant body weight (BW) reduction. In contrast, Laurindo et al. (9) reported that the same period of high-intensity ST was effective in reducing BW and mitigating the adverse effects frequently associated with obesity in rodents, including hepatic steatosis.

Although aerobic exercise is often recommended for managing obesity and insulin resistance, ST induces specific physiological adaptations that make it a promising strategy, particularly in the context of obesity-associated MASLD (8). Unlike aerobic exercise, ST has the potential to preserve and increase muscle mass, which is essential for energy metabolism and glucose homeostasis, while also enhancing muscular strength and functionality - factors that are frequently impaired in obesity (10). Additionally, ST directly modulates lipid metabolism and reduces visceral adipocyte area, contributing to a less deleterious inflammatory profile (11). Given that obesity is characterized by chronic low-grade inflammation and insulin resistance, both of which are key mechanisms in the progression of MASLD, investigating whether ST can attenuate these processes through its metabolic and hormonal adaptations is highly relevant (11-13).

Previous studies have shown that ST can significantly reduce hepatic steatosis and improve insulin sensitivity, even in the absence of substantial weight loss (14,15). However, there are still gaps in the literature regarding the mechanisms by which ST directly affects the liver in experimental models of obesity. Therefore, this study aimed to investigate the effects of ST on hepatic fat deposition in HFD-induced obese rats, analyzing its impact on inflammation and insulin resistance. The hypothesis of this study was that ST improves inflammatory markers and obesity-associated insulin resistance, thereby promoting the attenuation of hepatic steatosis.

## Material and Methods

### Animal care

Thirty-six male Wistar rats (∼150 g; 30 days old) were obtained from the Central Animal House of the Federal University of Espírito Santo (Brazil). The animals were housed in individual cages with a controlled environment in terms of light (12 h light/dark cycle starting at 6 am), clean-air room temperature (24±2°C), and relative humidity (55±5%). The experimental protocol was performed in accordance with the “Guide for the care and use of laboratory animals”, published by the U.S. National Institutes of Health and the Ethics Committee on the Use of Animals of the Federal University of Espírito Santo, under protocol 54/2019.

### Experimental protocol

Rats were submitted to a 7-day acclimatization period and then randomized into two groups: a) SD, fed a standard diet, and b) HFD, fed a high-fat diet. SD animals were fed a diet with a caloric density of 2.92 kcal/g, which contained 30.2% calories from protein, 55.9% from carbohydrates, and 13.9% from lipids (Nuvilab CR1-Nuvital, Brazil). HFD animals received a cycle of 4 alternating diets containing different flavoring additives, such as: cheese, chocolate, bacon, and vanilla for palatability and higher food consumption. This diet had a caloric density of 3.65 kcal/g, with 21.9% of its calories coming from protein, 28.9% from carbohydrates and 49.2% from lipids (RC Focus 2413, 2414, 2415, and 2416, Agroceres, Brazil). The nutritional composition of the experimental diets is presented in Table 1.

**Table 1:** Nutritional composition of experimental diets.

Components	Experimental groups	
	SD	HFD
Protein (g)	22.0	20.0
Carbohydrate (g)	40.8	26.4
Fat (g)	4.5	20.0
Vitamins and minerals (g)	12.2	12.1
Fiber (g)	8.0	9.0
Water content (%)	12.5	12.5
Calories (kcal/g)	2.92	3.65
Calories from protein (%)	30.2	21.9
Calories from carbohydrates (%)	55.9	28.9
Calories from fat (%)	13.9	49.2

Composition of diets in 100 g of feed. SD: standard diet; HFD: high-fat diet.

All animals had free access to water and were offered 40 g of pelleted feed per day; the remaining amount was measured after 24 h for subsequent calculation of caloric intake (daily food consumption = 40 g - amount of food not consumed). Caloric intake was calculated by multiplying daily food consumption by the caloric value of each diet (g × kcal). Feed efficiency was calculated by dividing the total weight gain of the animals (g) by the total energy ingested (kcal).

**Figure 1:**
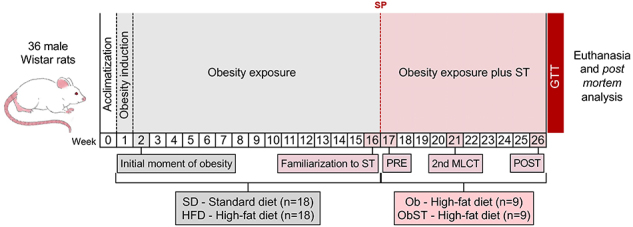
Schematic representation of the experimental protocol. SD: standard diet; HFD: high-fat diet; Ob: obese sedentary; ObST: obese sedentary submitted to strength training (ST); PRE: baseline maximum load-carrying test data; POST: final maximum load-carrying test; 2nd MLCT: 2nd maximum load carrying test; GTT: glucose tolerance test; SP: separation point for characterization of groups.

The protocol consisted of 2 experiments, which lasted 26 weeks: experiment 1 consisted of dietary submission and obesity induction and exposure (initial week to week 16), and experiment 2, where the groups were redistributed and the ST protocol was performed (week 17 to 26) (Figure 1).

### Obesity induction and exposure

Obesity induction lasted for 1 week and was characterized by increased BW in the HFD group compared to the SD group. This experimental period has been called the initial moment of obesity (16). Obesity was maintained for 15 weeks (week 2 to week 16), an important period for determining the duration of obesity.

At the end of the 16th week, a 95% confidence interval based on the mean BW from the SD and HFD rats was adopted as the cutoff point for the characterization of groups (16) as SD (n=18) and HFD (n=18). BW, total body fat (BF: epididymal fat + retroperitoneal fat + visceral fat), and adiposity index (AI: (total body fat (BF) / final BW) × 100) were measured to assess obesity.

### Obesity exposure and ST

After group separation based on the cutoff point, the HFD group was systematically randomized and renamed into two groups: obese sedentary (Ob, n=9) and obese sedentary submitted to ST (ObST, n=9).

The ST protocol, adapted from Hornberger Jr. and Farrar (10), consisted of voluntary training carried out on a ladder adapted for rats. The ladder was 110 cm high and 18 cm wide, with 2 cm between steps and 80° inclination. At the top of the ladder, there was a 20×20×20 cm resting box. The loads used in the training were made with ore powder packed in plastic bags attached to a fishing hook, which were attached to the proximal part of the animals' tail with 25-mm self-adhesive crepe tape. The characterization of the series was determined by the animal's voluntary climb, from the beginning of the steps until the complete entry into the resting box. The animals were encouraged to climb with food and by tapping on the animal's back and/or on the steps (11).

#### Familiarization to strength training

In the 16th week of the experimental protocol, the Ob and ObST groups were familiarized with the ST ladder protocol for three non-consecutive days (Monday, Wednesday, and Friday), without load. The animals were encouraged to perform four complete climbs up the stairs, with a 60 s rest interval between them. Before the first attempt, the rats were placed in the resting box to establish initial contact between the animal and the training apparatus. Afterwards, the animals were encouraged to climb progressively from the bottom to the top, four times (11).

#### Maximum load-carrying test

In the 17th week, the Ob and ObST animals performed the maximum load-carrying test (MLCT). Three tests were performed during the ST period: at baseline (PRE) in the 17th week, at the 21st week, and at the 26th week (POST). The animals began the PRE test carrying a load corresponding to 50% of their BW. For each complete set performed, 30 g was added to the load, until the animal was no longer able to carry it to the top. The 2nd MLCT was performed only with the ObST animals to monitor strength evolution, with the training loads being adjusted in each session based on the last training session. The POST test started with 50% of the maximum load carried by the animal at the last training session. The interval between each set was 120 s for all tests. The analyzed parameters were: 1) absolute load (g); 2) relative load (absolute load/BW); and 3) strength delta (Δ), calculated by the formula: (POST - PRE × 100 / PRE), reported as percentage (11).

#### Strength training

The protocol started from the 17th week onwards with the ObST group. Training was carried out three times a week (Mondays, Wednesdays, and Fridays) for 10 weeks and consisted of four to five climbs (sets) with high and progressive intensity: 50, 75, 90, and 100% of the MLCT or the last training session. Having completed the fourth climb, a fifth series with the addition of 30 g to the maximum load of 100% was encouraged in order to monitor the evolution of strength and adjust the maximum load values throughout the protocol. The recovery interval between sets was 60 s (11).

### Glucose tolerance test

After the completion of the experimental protocol, the animals underwent a 6-h fasting period, after which blood samples were collected from the caudal artery under basal conditions and following the intraperitoneal administration of a 25% glucose solution (2 g/kg BW) at 30, 60, 90, and 120 min (17). The glucose tolerance was assessed by the area under the curve (AUC) for glucose using the formula (18): AUC = [(glucose basal) + (30' × 2) + (60' × 3) + (120' × 2)] / 4.

### Euthanasia

Euthanasia was performed on a different day than the glucose tolerance test and 48 h after the last maximum load-carrying test. The animals fasted for 12 to 15 h and then received an intraperitoneal (*ip*) injection of sodium heparin (1000 U/kg). After 30 min, rats were anesthetized with ketamine (70 mg/kg, *ip*, Dopalen; Sespo Indústria e Comércio Ltda., Vetbrands Division, Brazil) and xylazine (10 mg/kg, *ip*; Anasedan; Sespo Indústria e Comércio Ltda., Vetbrands Division) and then euthanized. A deep anesthetic plane was ensured prior to euthanasia, and a single higher dose (20-30% of the initial dose of anesthetics) was administered when needed to ensure the absence of nociception. After euthanasia, the animals were submitted to median thoracotomy for blood and tissue sample collection.

### Biochemical and hormonal profiles

Plasma concentrations of glucose, total cholesterol (TC), triglyceride (TG), high-density lipoprotein cholesterol (HDL), alanine aminotransferase (ALT), and aspartate aminotransferase (AST) were determined using specific kits (Bioclin Bioquímica^®^ and Synermed do Brasil Ltda., Brazil) and analyzed by the automated biochemical analyzer. Plasma concentrations of IL-6 (Invitrogen, Thermo Fisher Scientific, USA), TNF-α (Invitrogen, Thermo Fisher Scientific), insulin (Millipore, Sigma Aldrich, USA), adiponectin (Millipore, Sigma Aldrich), and leptin (Linco Research Inc., USA) were quantified by the enzyme-linked immunosorbent assay method (ELISA). The adiponectin/leptin ratio (A/L) was calculated according to Frühbeck et al. (19) and the homeostatic model assessment index (HOMA-IR), as proposed by Matthews et al. (20).

### Determination of liver water content

Liver samples were dried in a micro-processed drying oven (Quimis, Q317M2, Brazil) at 55±5°C for 48 h. The determination of the water content was reported in relative values and calculated by the following formula: [(fresh weight (g) - dry weight (g)) / (fresh weight (g)) × 100].

### Morphological analysis

Frozen retroperitoneal and visceral (mesenteric) fat pads and liver samples were thawed and fixed in 4% paraformaldehyde. The samples were dehydrated in ethanol, clarified in xylene, and embedded in paraffin. Paraffin blocks were cut into 5-µm-thick sections and were stained with hematoxylin and eosin (H&E). The images were captured with a 10× (adipose tissue) and 40× (liver tissue) magnification using a video camera (LAS EZ^®^, ICC50 HD, 51112061; Leica, Germany) coupled to an optical microscope (Leica, RM 2125 RTS).

Assessment of fat deposits included the analysis of adipocyte area and number. The adipocyte area measurement was performed using ImageJ software (National Institutes of Health, USA). The adipocyte area was calculated for each group from the average value of the area in all fields measured. The adipocyte number was calculated according to Gibert-Ramos et al. (21). H&E-stained liver sections were evaluated for inflammation and steatosis scores, both classified on a scale of 0-3, as proposed by Müller et al. (22): 0) no changes; 1) slight changes; 2) stronger changes; 3) intense changes.

To evaluate hepatic fibrotic area, paraffin blocks were cut into 5-µm-thick sections and stained with Sirius red. Images were captured at 20× magnification, and the percentage of the fibrotic area was measured using ImageJ software.

Frozen liver samples were embedded in Tissue Plus O.C.T. Compound (Fisher HealthCare, Thermo Fisher Scientific, USA), sectioned at 10-µm-thickness using a cryostat at -25°C (Jung CM 1860; Leica), and then stained in Oil Red-O (Sigma Aldrich). The images were captured at a 40× magnification and semiquantitative analysis of the percentage of steatosis was measured using ImageJ software.

### Pancreas immunohistochemistry

After being fixed in 4% paraformaldehyde, pancreas samples were processed and embedded in paraffin. Paraffin blocks were cut into 3-µm-thick sections using a microtome and submitted to immunohistochemistry (Spring Bioscience^®^ PMB1-250, USA). Peroxidases and endogenous proteins were blocked and then the samples were incubated with polyclonal primary antibody insulin (1:100, Cell Signaling Technology, USA). Reaction amplification was then induced by universal immunoperoxidase polymer (Spring Bioscience^®^ PMB1-250) for 30 min. Sections were washed, and the colorimetric reaction was developed with DAB (Spring Bioscience^®^ PMB1-250) and counterstained with hematoxylin. Insulin-immunoreactive cells were stained with brown staining in the cytoplasm of β cells. The images were captured with a 40× magnification, using a video camera (LAS EZ^®^, ICC50 HD - 51112061, Leica) coupled to an optical microscope (Leica, RM 2125 RTS). Images of all islets of the immunostained samples were identified and measurement was performed using ImageJ software. The analyzed parameters were: 1) number of pancreatic β cells (number of nuclei surrounded by insulin immunostaining) and 2) size of pancreatic β cells (insulin-positive area divided by the number of nuclei of immunostained cells).

### Statistical analysis

The Shapiro-Wilk normality test was used for data distribution. Data are reported as means±SD. Comparisons between groups were performed using the Student's *t*-test for independent samples. BW evolution was evaluated using two-way repeated measures ANOVA, complemented with the *post hoc* Bonferroni test. The association between single linear variables was explored by Pearson's correlation coefficient. Effect size (Cohen's d) was employed to quantify the standardized mean difference associated with the effects of obesity or ST. All analyses and graphs were created using the GraphPad Prism 8.4.3 software (USA). Statistical conclusions were discussed based on a significant level of 5%.

## Results

### Obesity induction and exposure

At the beginning of the experimental protocol, the animals in the SD and HFD groups had similar BW. The animals received their respective diets and after the obesity induction period, the HFD group presented a statistically higher BW than the SD group (P=0.0006, effect size 1.06) at the 2nd week of treatment, thus characterizing the initial moment of obesity. Subsequently, HFD animals underwent the 2nd to 16th week of the obesity exposure period, exhibiting a significantly greater body weight gain compared to the SD group (P<0.0001, effect size 2.08). There was no statistical difference between the groups in food consumption. In contrast, caloric intake (P<0.0001, effect size 2.07) and feed efficiency (P=0.0232, effect size 0.76) of the HFD group were higher compared to the SD group (HFD>SD) (Table 2).

**Table 2:** Nutritional profile and body weight (BW) gain during obesity induction and exposure periods (weeks 1 to 16).

Variables	Experimental Groups	
	SD	HFD
Food consumption (g/day)	22.0±1.9	21.3±2.0
Caloric intake (kcal/day)	64.3±5.4	77.7±7.4*
Feed efficiency (%)	4.54±0.28	4.73±0.23*
IBW (g) 0 week	126±19.5	129±13.6
BW (g) 16th week	455±37.2	541±43.7*
BW gain (g) 0-16th week	329±38.0	412±41.5*

Data are reported as means±SD. SD: standard diet (n=18); HFD: high-fat diet (n=18); BW: body weight; IBW: initial body weight. *P<0.05, Student's *t*-test.

**Figure 2:**
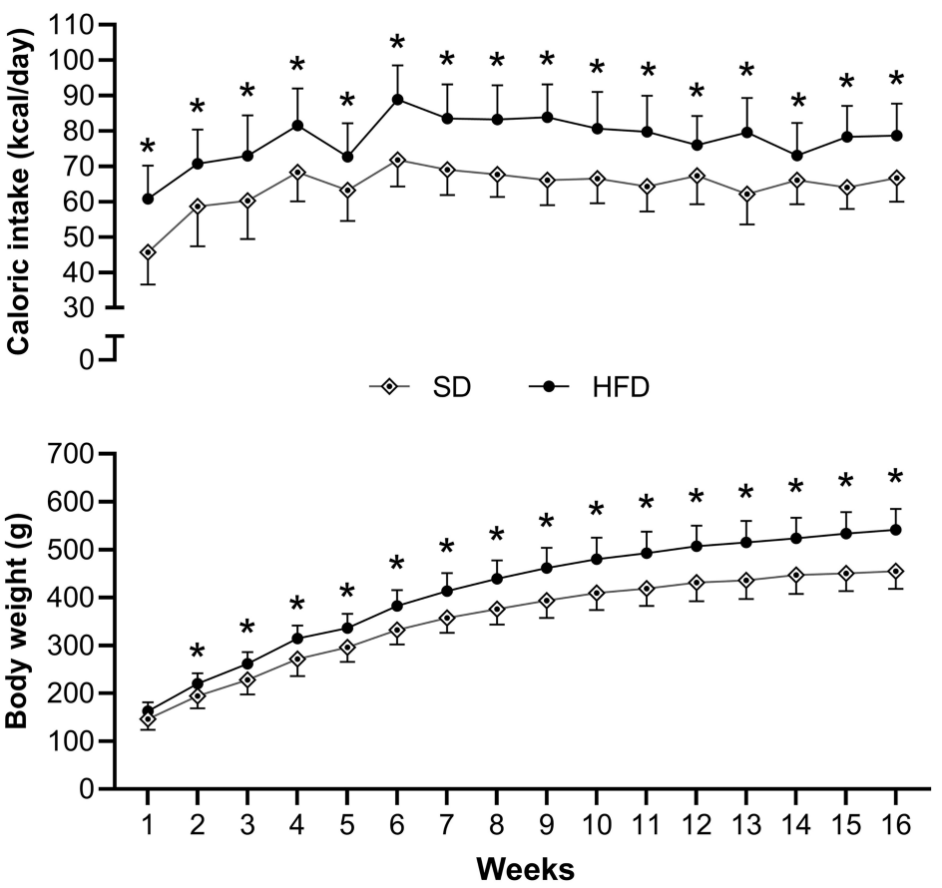
Evolution of caloric intake and body weight during obesity induction and exposure periods (weeks 1 to 16). Data are reported as means±SD. *P<0.05, two-way ANOVA for repeated measures, supplemented with *post hoc* Bonferroni test. SD: standard diet (n=18); HFD: high-fat diet (n=18).

Figure 2 shows the evolution of caloric intake and BW of SD and HFD animals. The HDF animals ingested more calories than the SD animals and maintained a statistically higher BW from the 2nd week onwards. In this context, the BW gain in the HFD group may be explained by the higher caloric value of the HFD, which had an energy density of 3.65 kcal/g, while the SD diet had an energy density of 2.92 kcal/g.

### Obesity exposure plus ST

**Figure 3:**
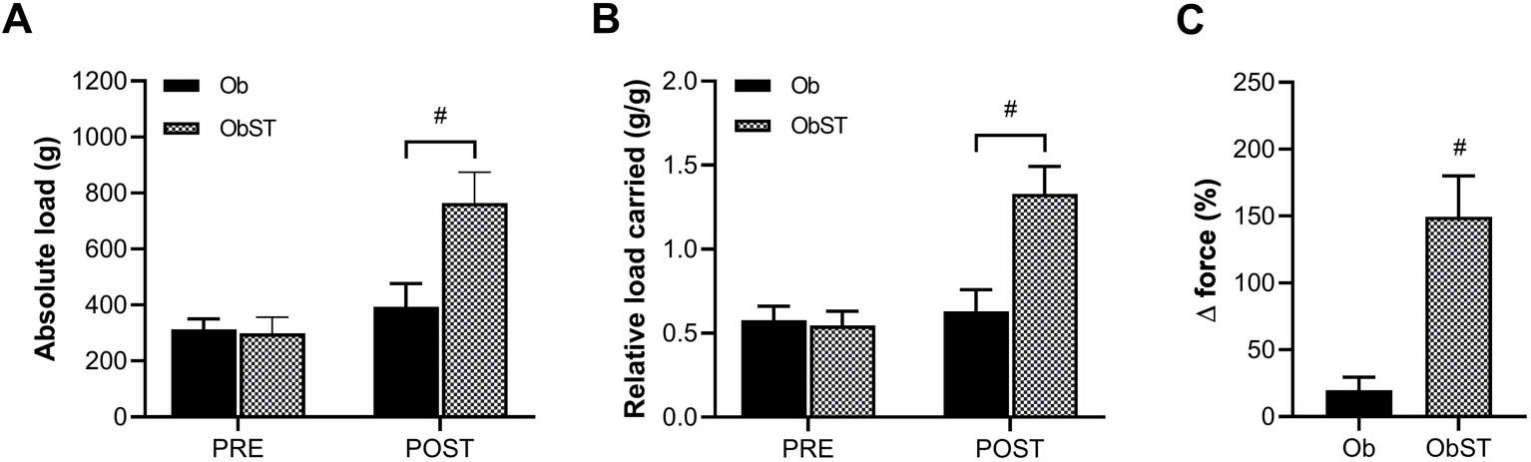
Maximum load-carrying test data at baseline (PRE) and at the end of the protocol (POST). **A**, Absolute load. **B**, Relative load carried. **C**, Delta force (Δ force). Data are reported as means±SD. ^#^P<0.05, two-way ANOVA for repeated measures, supplemented with *post hoc* Bonferroni test (PRE and POST) and Student's *t*-test (Δ force). Ob: obese sedentary (n=9); ObST: obese sedentary submitted to strength training (n=9).

There were no statistical differences in absolute and relative training loads among groups in PRE training (Figure 3A and B). ST promoted an elevation of absolute and relative loads in POST training compared with Ob (absolute load P<0.0001, effect size 3.81; relative load P<0.0001, effect size 4.72). Likewise, the results of Δ force were also statistically higher in the trained group (P<0.0001, effect size 5.75) (Figure 3C).

**Figure 4:**
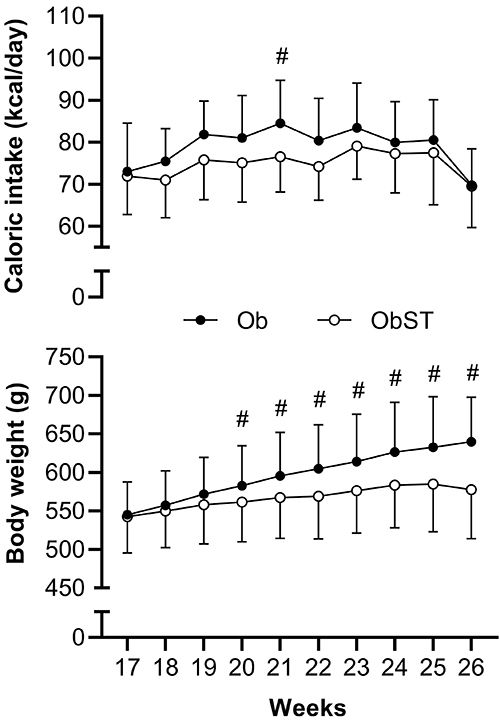
Evolution of caloric intake and body weight during obesity exposure plus strength training period (weeks 17 to 26). Data are reported as means±SD. ^#^P<0.05, two-way ANOVA for repeated measures, supplemented with *post hoc* Bonferroni test. Ob: obese sedentary (n=9); ObST: obese sedentary submitted to strength training (n=9).

Figure 4 shows the evolution of caloric intake and BW of Ob and ObST groups. With the exception of week 21, there were no differences in caloric intake between the groups. On the other hand, from the 20th week onwards, ST promoted less BW gain in ObST animals compared to untrained animals (ObST<Ob). In this sense, ST attenuated the effect of HFD, preventing excessive BW gain during the 10 weeks of exercise.

ST promoted a reduction in feed efficiency (P=0.0005, effect size 2.06), BW gain (P=0.0005, effect size 2.07), final body weight (FBW; P=0.0452, effect size 1.02), epididymal fat (P=0.0023, effect size 1.71), visceral fat (P=0.0112, effect size 1.35), total body fat (P=0.0164, effect size 1.26), and AI (P=0.0210, effect size 1.21). However, ST did not promote changes in food consumption, caloric intake, or retroperitoneal fat pads (Table 3).

**Table 3:** Effect of strength training on nutritional parameters and adiposity characteristics of obese rats.

Variables	Experimental Groups	
	Ob	ObST
Food consumption (g/day)	21.63±2.33	20.5±2.22
Caloric intake (kcal/day)	79.0±8.50	74.8±8.10
Feed efficiency (%)	1.72±0.53	0.64±0.51^#^
BW (g) 17th week	545±42.7	542.5±46.8
FBW (g) 26th week	640±58	577.7±63.7^#^
BW gain (g) 17th-26th week	95±27.7	35.2±30.1^#^
Epididymal fat (g)	15.6±4.57	9.81±1.43^#^
Retroperitoneal fat (g)	28.2±7.14	22.6±6.42
Visceral fat (g)	18.6±5.91	12.0±3.45^#^
Body fat (g)	62.4±17.1	44.4±10.6^#^
Adiposity index (%)	9.81±1.74	7.64±1.27^#^

Data are reported as means±SD. ^#^P<0.05; Student's *t*-test. Ob: obese sedentary (n=9); ObST: obese sedentary submitted to strength training (n=9). BW: body weight; FBW: final body weight; Body fat: sum of epididymal, retroperitoneal, and visceral fats.

Regarding metabolic changes, ST promoted a reduction of TC (P=0.0335, effect size -1.10), TG (P=0.0010, effect size 1.89), AST (P=0.0329, effect size 1.10), baseline glucose (P=0.0066, effect size 1.47), HOMA-IR (P=0.0382, effect size 1.15), leptin (P=0.0024, effect size 1.70), and TNF-α plasma levels (P=0.0445, effect size 1.03) (ObST<Ob), as well as an increased adiponectin/leptin ratio (A/L) (P=0.0076, effect size 1.44). However, it did not significantly change the other variables (Table 4).

**Table 4:** Biochemical, glycemic, hormonal, and inflammatory profiles.

Variables	Experimental Groups	
	Ob	ObST
TC (mg/dL)	62.2±6.91	54.1±7.88^#^
HDL (mg/dL)	25.3±2.04	24.9±4.48
TG (mg/dL)	31.2±6.66	20.6±4.42^#^
ALT (U/L)	62.9±11.8	55.4±7.50
AST (U/L)	164±65.6	111±18.9^#^
Glucose (mg/dL)	115±2.97	104±10.3^#^
AUC (mg/dL/min)	271±49.0	260±50.6
Insulin (ng/mL)	2.15±1.23	1.25±0.54
HOMA-IR	17.4±9.67	9.27±4.19^#^
Leptin (ng/mL)	19.0±4.96	10.8±4.67^#^
Adiponectin (ng/mL)	4.10±0.44	4.25±0.49
A/L	0.23±0.07	0.47±0.22^#^
IL-6 (pg/mL)	16.1±1.64	14.8±2.17
TNF-α (pg/mL)	67.1±15.9	54.9±5.56^#^

Data are reported as means±SD. ^#^P<0.05, Student's *t*-test. Ob: obese sedentary (n=9); ObST: obese sedentary submitted to strength training (n=9). TC: total cholesterol; HDL: high-density lipoprotein; TG: triglycerides; ALT: alanine aminotransferase; AST: aspartate aminotransferase; AUC: area under the curve for glucose; HOMA-IR: homeostatic model assessment index; A/L: adiponectin/leptin ratio; IL-6: interleukin-6. TNF-α: tumor necrosis factor-alpha.

**Figure 5:**
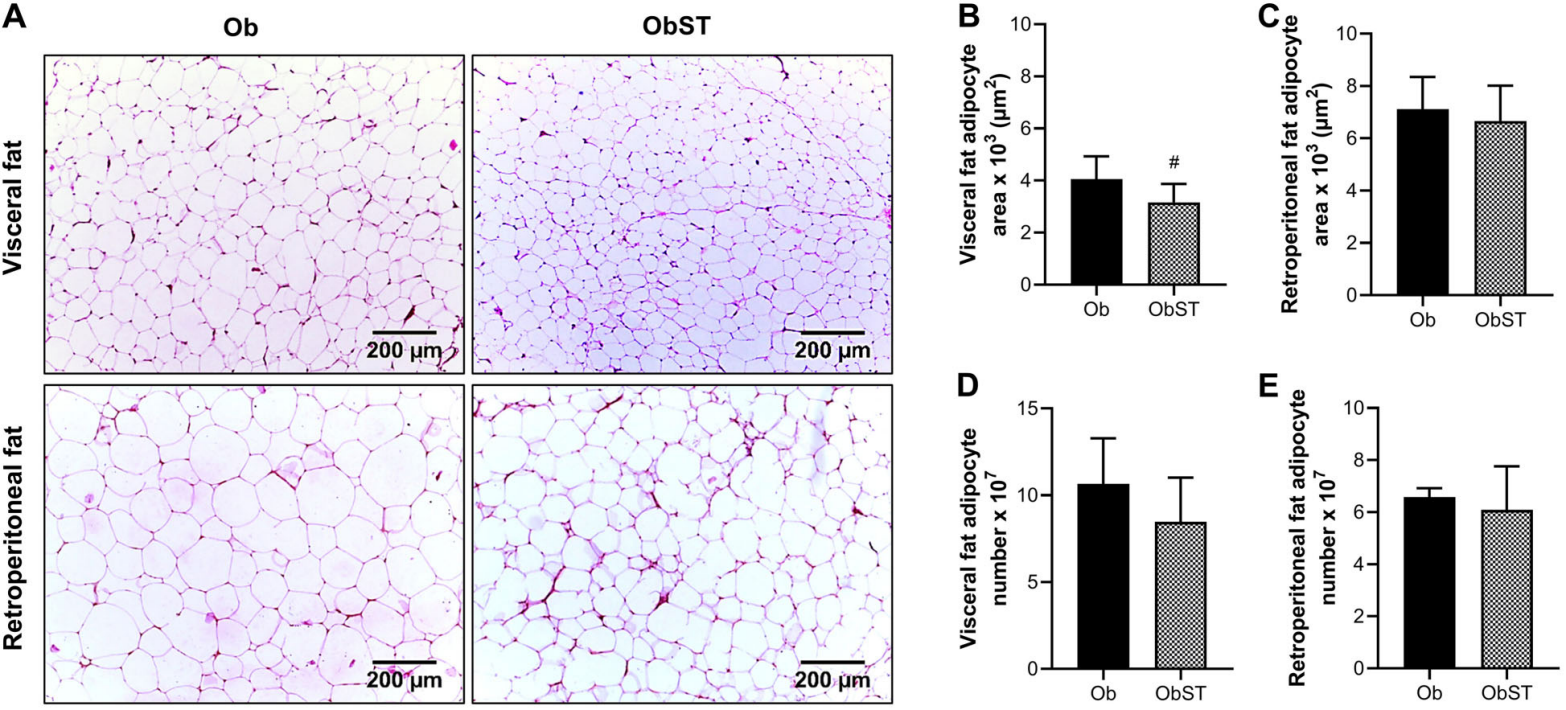
Histological analysis of the adipose tissues. **A**, Representative photomicrographs of retroperitoneal and visceral fat fragments, stained in H&E (10× magnification; scale bar: 200 μm). **B**, Visceral and (**C**) retroperitoneal fat adipocytes area. **D**, Visceral and (**E**) retroperitoneal fat adipocytes number. Data are reported as means±SD. ^#^P<0.05, Student's *t*-test. Ob: obese sedentary (n=9); ObST: obese sedentary submitted to strength training (n=9).

**Figure 6:**
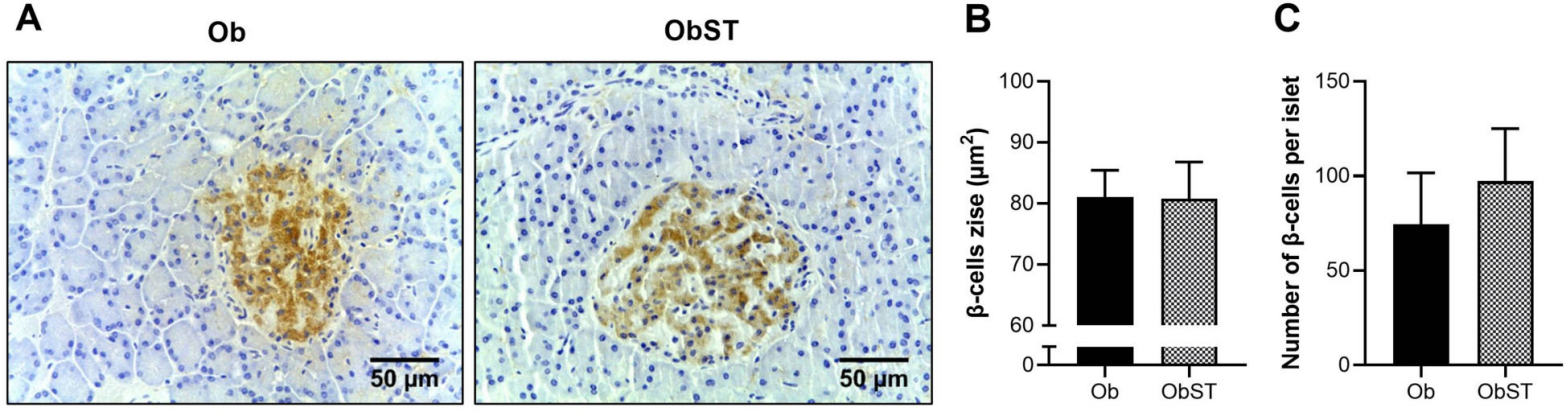
Effect of strength training on pancreas histology. **A**, Representative photomicrographs of the immunohistochemical reaction with insulin (brown), contrasted with hematoxylin (40× magnification; scale bar: 50 μm). **B**, β-cells size (area). **C**, Number of β-cells per pancreatic islet. Data are reported as means±SD, P>0.05, Student's *t-*test. Ob: obese sedentary (n=5); ObST: obese sedentary submitted to strength training (n=5).

There were no statistical differences in the number of adipocytes in visceral and retroperitoneal fat pads (Figures 5D and E). The retroperitoneal fat adipocyte area also did not differ between groups (Figure 5C). However, the visceral fat adipocyte area was lower in the ObST group (P=0.0363, effect size -1.12) compared to Ob rats (Figure 5B), indicating a reduction in visceral adipocyte size in response to the training protocol, which may reflect adaptations in fat storage dynamics and remodeling of adipose tissue. In the present study, the absence of changes in plasma insulin levels was in agreement with the immunohistochemical analysis of the pancreas since ST did not change the area and number of pancreatic β cells (Figure 6).

**Figure 7:**
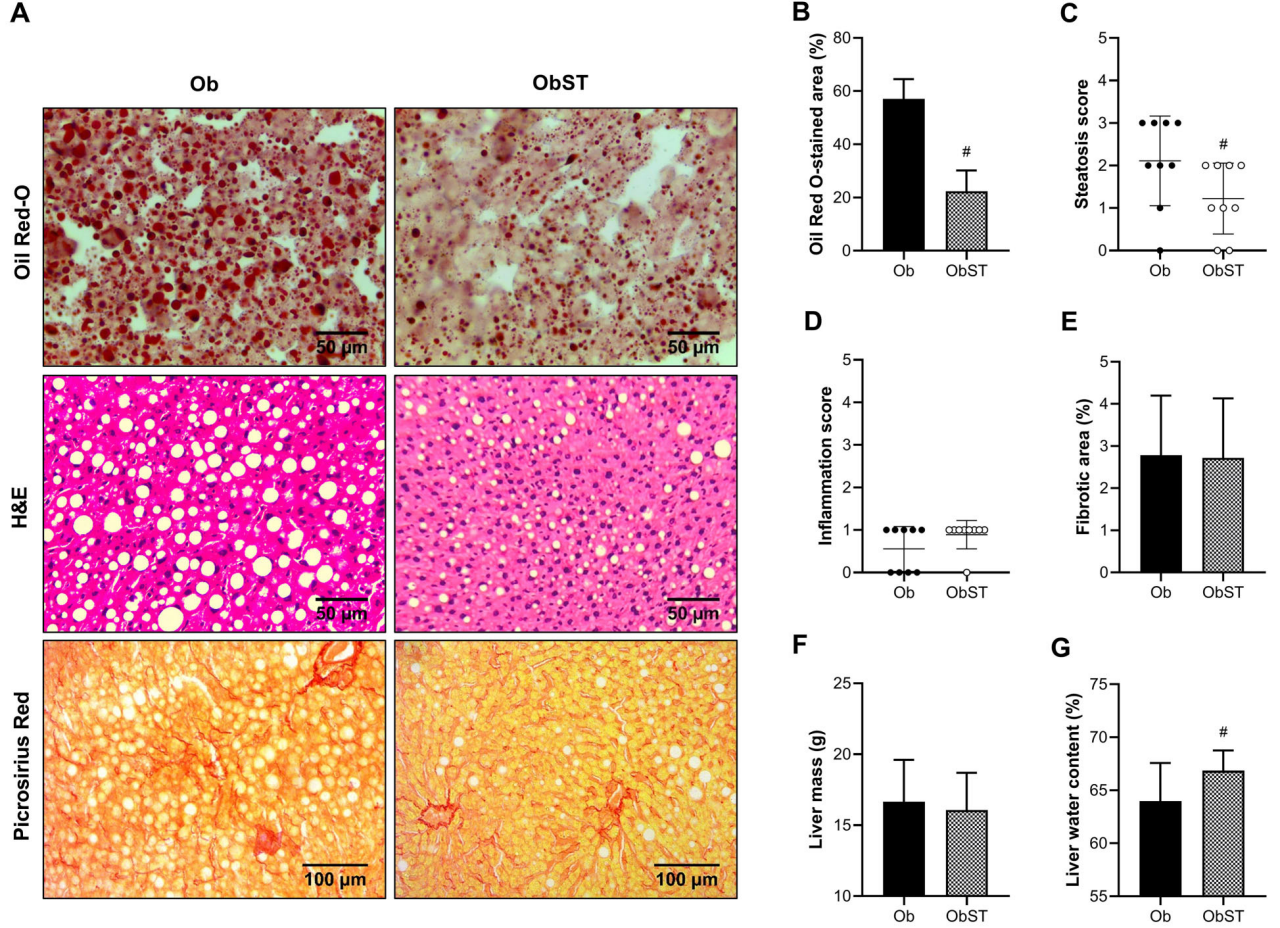
Evaluation of the liver tissue. **A**, Representative photomicrographs of liver tissue, stained with Oil Red-O (40× magnification), H&E (40× magnification), and Picrosirius Red (20× magnification; scale bars: 50 μm; 100 μm. **B**, Hepatic steatosis area by Oil Red-O analysis. **C**, Grading score of hepatic steatosis. **D**, Grading score of liver inflammation. **E**, Fibrotic area in the liver by Sirius Red analysis. **F**, Liver mass. **G**, Liver water content. Data are reported as means±SD. ^#^P<0.05, Student's *t*-test. Ob: obese sedentary (n=9); ObST: obese sedentary submitted to strength training (n=9).

As shown in Figure 7, liver sections from the ObST group had fewer fat microdroplets (P=0.0440, effect size 1.2), demonstrating that ST was effective in reducing the degree of steatosis (Figure 7C). Corroborating the result, Oil Red-O-stained liver sections showed a smaller hepatic steatosis area in ObST animals (P<0.0001, effect size 4.58) (Figure 7B). No statistical difference was observed regarding the magnitude of MASLD since there were no inflammatory infiltrates and fibrotic areas in the liver tissue (Figures 7D and E). No statistical difference was observed in liver mass (Figure 7F), however, liver water content was higher in the presence of ST (P=0.0497, effect size 1.00) (Figure 7G).

**Figure 8:**
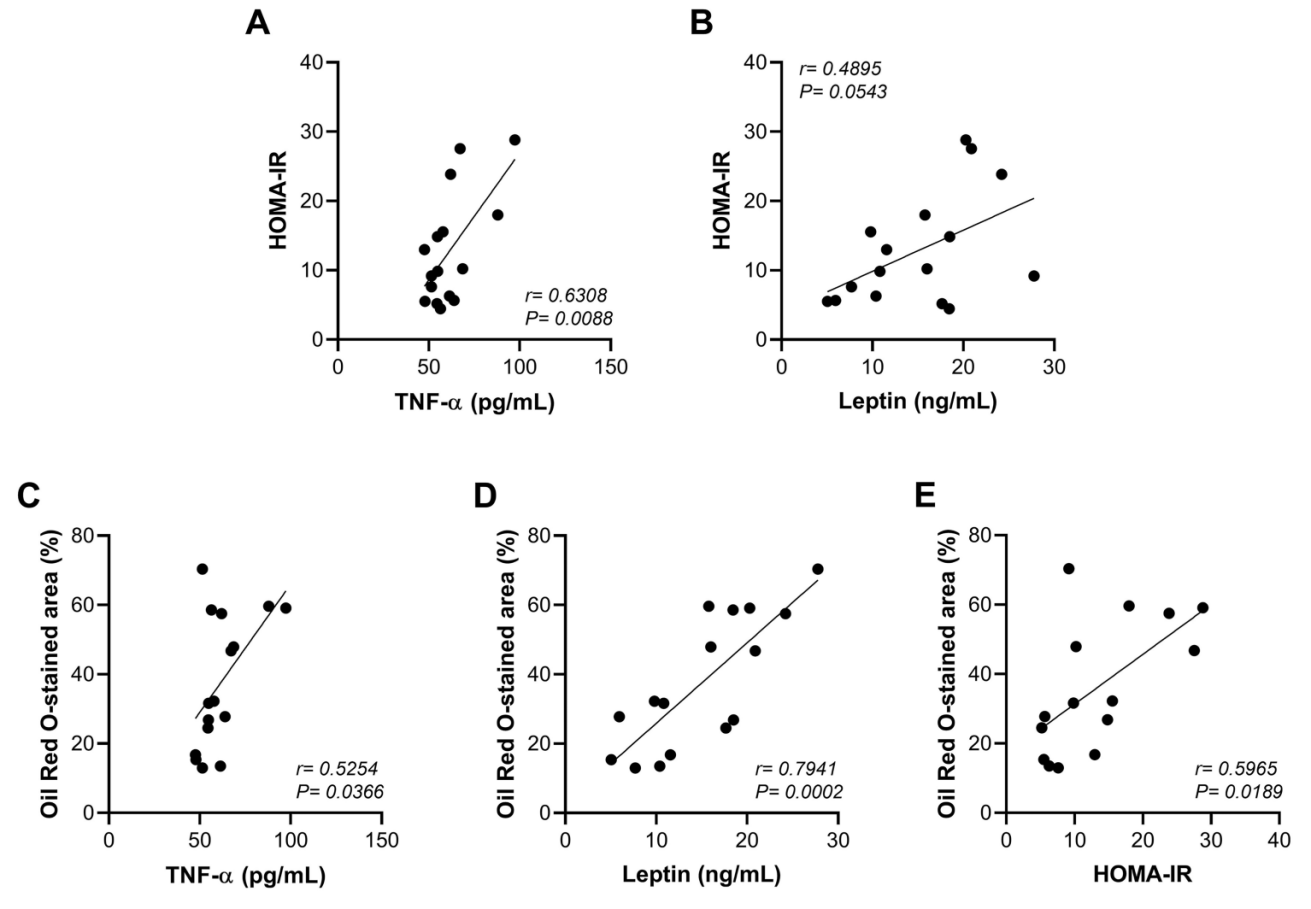
Pearson's correlations between hepatic steatosis area and strength training-induced metabolic changes in obesity. Correlation analyses between **A**, insulin resistance (IR) and TNF-α; **B**, IR and leptin; **C**, hepatic steatosis area and TNF-α; **D**, hepatic steatosis area and leptin; and **E**, hepatic steatosis area and IR. TNF-α: tumor necrosis factor-alpha; HOMA-IR: homeostatic model assessment index. Data are reported as means using correlation coefficient. Ob: obese sedentary (n=7); ObST: obese sedentary submitted to strength training (n=9).

Correlation analyses between insulin resistance and inflammatory biomarkers are shown in Figure 8. A moderate positive correlation was observed between insulin resistance and TNF-α (r=0.6308, P=0.0088) (Figure 8A). However, leptin had a weak positive correlation with insulin resistance (r=0.4895, P=0.0543) (Figure 8B).

A strong positive correlation was observed between hepatic steatosis area (under the Oil Red-O analysis) and leptin (r=0.7941, P=0.0002) (Figure 8D). A moderate positive correlation was observed between the steatosis area and TNF-α (r=0.5254, P=0.0366) and insulin resistance (r=0.5965, P=0.0189) (Figures 8C and E).

## Discussion

The main findings of the current study indicated that ST in the condition of obesity promoted reduction of visceral fat adipocyte area and improvements in the lipid and glycemic profiles, insulin resistance, and inflammatory biomarkers. ST was effective in attenuating fatty liver in a model of obesity induced by a HFD, favoring a 34.7% reduction in hepatic steatosis. In this context, ST effectively reduced the hepatic steatosis graduation score, being classified as “stronger changes” in the obesity condition (score 2.22) and as “slight changes” (score 1.22) in obesity + training. According to Baek et al. (8), even with HFD intake, exercise independently improves hepatic lipid content.

The dietary intervention consisted of the use of a HFD containing 49.2% of its calories from lipids, which corresponded predominantly to unsaturated fatty acids. The HFD was of sufficient intensity and duration to promote obesity after 26 weeks. The data showed that, from the 2nd week, the BW of the HFD group was higher than the SD group. According to Buettner et al. (23), the difference in BW induced by a HFD can be observed from the 2nd week of treatment but becomes more apparent after 4 weeks. Previous studies, which used the same dietary composition and obesity characterization criteria, had observed a difference in the BW from the 3rd week of treatment, which was also maintained until the end of the protocol (11,16).

In the current study, the daily food consumption remained the same in the two groups. Considering that lipids are the most caloric component of the diet and provide 9 kcal/g, food satiety can be achieved without the animals necessarily having to ingest large amounts of food (24). In this sense, BW gain in the Ob group can be explained by the increase in caloric intake from the HFD, which had a higher energy density compared to the SD (3.65 *vs* 2.92 Kcal/g). In a previous study from our laboratory, the diet favored an increase in feed efficiency, indicating a greater capacity of the HFD animals to convert consumed energy into BW (16).

In the physical performance analyses, the loads carried, both absolute and relative to BW, did not differ between the groups in the maximum load-carrying test before training, indicating that the animals had similar strength levels at baseline. However, in the post-training test, ObST animals carried significantly greater absolute and relative loads compared to sedentary Ob animals, demonstrating that the ST protocol effectively increased muscle strength. These findings are consistent with previous studies that applied high-intensity ladder ST for 10 weeks (11,17), showing that ObST animals exhibited improved load-carrying capacity. In ST models, the relative load carried is considered a reliable indicator of functional performance, as it reflects adaptations in muscle strength and endurance (25). In the present study, the improvements in muscle strength observed in ObST animals contributed to mitigating the negative impact of obesity on physical performance, reinforcing the benefits of ST in counteracting obesity-related functional impairments.

The ladder ST protocol promoted lower BW gain in ObST animals from the 19th week of treatment (3rd week of ST) compared to sedentary Ob animals. Lower BW gain was accompanied by lower adiposity. ObST animals presented lower visceral fat adipocyte area. This result can be explained, in part, to their greater sensitivity to catecholamines and greater expression of β adrenergic receptors, and thus, greater susceptibility to lipolysis (26). In contrast, retroperitoneal fat has a lower expression of β adrenergic receptors and, therefore, less susceptibility to lipolysis (26), which would explain the absence of change in the retroperitoneal fat adipocyte area.

In obesity, adipose tissue hypertrophy leads to macrophage infiltration and the production of pro-inflammatory cytokines, such as TNF-α and IL-6, which play central roles in chronic inflammation and insulin resistance (27). In this study, we observed a significant reduction in plasma TNF-α concentrations in the trained group, suggesting an anti-inflammatory effect of the ST protocol. TNF-α is one of the key mediators of obesity-associated inflammation and can inhibit insulin signaling by activating the JNK/NF-κB pathways, impairing insulin receptor phosphorylation, and contributing to hepatic lipotoxicity (28). Thus, the reduction in TNF-α observed in the ObST group may have contributed to improved glycemic homeostasis and insulin sensitivity, thereby reducing hepatic lipid accumulation.

Conversely, IL-6 levels did not differ significantly between groups, indicating that ST, under the conditions of this study, did not modulate the systemic expression of this cytokine. IL-6 has a dual role, acting as a pro-inflammatory cytokine when secreted by adipose tissue macrophages and as an anti-inflammatory myokine when released by skeletal muscle in response to exercise (29). In the context of exercise, its transient release can contribute to beneficial metabolic effects, such as enhanced glucose uptake and lipid oxidation (27). However, the absence of significant differences in serum IL-6 levels suggests that the intensity and volume of ST applied in this study may not have been sufficient to elicit a notable response in this cytokine. Additionally, studies indicate that the IL-6 response to exercise is more pronounced in continuous aerobic training protocols of longer duration, whereas in ST, the magnitude of the response may be influenced by factors such as volume, load, and recovery time between sets (29). Considering our findings (reduction in TNF-α without alterations in IL-6), we can suggest that ST may have partially attenuated the obesity-associated inflammatory state without impacting the secretion of pro-metabolic myokines.

In this study, we also observed a significant reduction in plasma leptin concentrations in the trained group, which can be attributed to the decrease in total adiposity (30). Since leptin is synthesized and secreted by adipocytes in proportion to fat stores, its reduction was expected due to the lower adipose tissue mass in animals subjected to ST (11,17). In this context, the reduction in body fat induced by ST was responsible for decreasing leptin concentration by 43% compared to the sedentary Ob group. However, despite being a hormone involved in food intake regulation, no significant differences in food consumption were observed between groups. This suggests that leptin reduction did not impair energy homeostasis, possibly due to improved hypothalamic leptin sensitivity (13). Literature indicates that obesity is associated with leptin resistance, a condition in which the hormone loses its ability to inhibit appetite and increase energy expenditure (13). Chronic inflammation in obesity contributes to this condition, as TNF-α and the suppressor of cytokine signaling 3 (SOCS3) pathways impair leptin signaling in the hypothalamus (31). Thus, the reduction in TNF-α observed in the trained group may have contributed to an improved hypothalamic response to leptin, supporting metabolic homeostasis without compensatory effects on food intake.

Additionally, adipose tissue analysis revealed that ST increased the adiponectin/leptin (A/L) ratio, a widely used biomarker for assessing adipose tissue functionality (32). Adiponectin has anti-inflammatory and insulin-sensitizing properties, and its inverse relationship with leptin has been proposed as a more robust indicator of metabolic function than the absolute levels of each hormone (19,32). The increased A/L ratio in the trained group suggests adipose tissue remodeling toward a more functional phenotype, which may be associated with enhanced lipolytic capacity and greater utilization of fatty acids as an energy source. Furthermore, elevated leptin levels have been linked to the worsening of hepatic steatosis, as leptin activates the JAK2/STAT3 pathway, increasing the expression of the suppressor of SOCS3, which contributes to hepatic lipid accumulation (31,33). In the current study, the strong positive correlation between leptin levels and hepatic steatosis area reinforces this pathophysiological mechanism, suggesting that leptin reduction may have directly contributed to lower hepatic lipid deposition.

Although quantifying protein expression in the leptin signaling pathway in the brain would be a relevant approach to directly confirm these influences, our findings suggest that improvements in the A/L ratio, reduction of TNF-α levels, and decreased visceral fat deposition may be associated with restored leptin sensitivity, contributing to metabolic and inflammatory regulation in obesity.

Pereira et al. (14) showed a reduction in hepatic lipogenesis linked to a reduction in insulin resistance in ObST animals. In the insulin resistance state, hyperinsulinemia and hyperglycemia act by increasing the expression of transcriptional factors (SREBP-1c and ChREBP) involved in the expression of lipogenic genes, which stimulate hepatic *de novo* lipogenesis (DNL), including sterol regulatory element binding protein 1c (SREBP-1c) and carbohydrate response element binding protein (ChREBP) (7). According to Krisan et al. (34), ST can act in reversing the insulin resistance state, increasing the phosphoinositide 3-kinase (PI3K) activity in the insulin signaling pathway. In addition, according to Carson (35), during skeletal muscle contraction, upregulation of glucose transporter 4 (GLUT-4) can occur through the activation of AMP-activated protein kinase (AMPK) and thus improve glucose metabolism. The reduction in fatty liver of ObST animals in the present study may suggest that ST attenuated hepatic DNL, with a reduction in HOMA-IR.

MASLD evolves progressively and therefore its prevention and treatment are of fundamental importance even in the early stages, which are reversible (15). Elevations in transaminase concentrations may represent liver damage (36), marked by degeneration or cell death (37). In the current study, ST in the condition of obesity promoted a reduction in AST, with no change in ALT concentration. Kim et al. (38) reiterate that in acute liver injury, AST levels are more pronounced, compared to ALT, due to higher enzyme activity in hepatocytes. However, no statistical difference was observed in liver inflammation scores and fibrotic area, possibly indicating the absence of metabolic dysfunction-associated steatohepatitis (MASH) in the animals in the present study (2,39). Sookoian and Pirola (40) suggest that enzymes should not be restricted to only markers of injury to hepatocytes; in this context, it is possible that the ST-induced transaminase alteration is associated with another non-investigated metabolic alteration.

In summary, ST was effective in attenuating hepatic steatosis in an experimental model of HFD-induced obesity. These beneficial effects were accompanied by improvements in insulin resistance and a reduction in plasma leptin and TNF-α concentrations, suggesting that these biomarkers play a relevant role in hepatic fat accumulation. The improvement in the A/L ratio in the trained group reinforces the hypothesis that ST promotes adipose tissue remodeling toward a more functional phenotype, enhancing lipid utilization as an energy source. Additionally, the observed increase in muscle strength and reduction in adiposity in the trained group highlight the metabolic benefits of RT, independent of weight loss.

### Study limitations

First, the protein expression of key signaling pathways involved in energy homeostasis, such as leptin signaling in the hypothalamus and AMPK activation in the liver, was not assessed, which could provide a more detailed understanding of the molecular mechanisms involved. Furthermore, the absence of significant changes in serum IL-6 levels suggests that the inflammatory response to ST may depend on the intensity, volume, and duration of the applied protocol, aspects that should be further explored in future studies. Another limitation was that this study was conducted in an animal model, and direct extrapolation of these findings to humans should be approached with caution.
